# The reactivation of somatosensory cortex and behavioral recovery after sensory loss in mature primates

**DOI:** 10.3389/fnsys.2014.00084

**Published:** 2014-05-12

**Authors:** Hui-Xin Qi, Jon H. Kaas, Jamie L. Reed

**Affiliations:** Department of Psychology, Vanderbilt UniversityNashville, TN, USA

**Keywords:** area 3b, cuneate nucleus, monkey, plasticity, spinal cord, ventroposterior nucleus, somatosensory system

## Abstract

In our experiments, we removed a major source of activation of somatosensory cortex in mature monkeys by unilaterally sectioning the sensory afferents in the dorsal columns of the spinal cord at a high cervical level. At this level, the ascending branches of tactile afferents from the hand are cut, while other branches of these afferents remain intact to terminate on neurons in the dorsal horn of the spinal cord. Immediately after such a lesion, the monkeys seem relatively unimpaired in locomotion and often use the forelimb, but further inspection reveals that they prefer to use the unaffected hand in reaching for food. In addition, systematic testing indicates that they make more errors in retrieving pieces of food, and start using visual inspection of the rotated hand to confirm the success of the grasping of the food. Such difficulties are not surprising as a complete dorsal column lesion totally deactivates the contralateral hand representation in primary somatosensory cortex (area 3b). However, hand use rapidly improves over the first post-lesion weeks, and much of the hand representational territory in contralateral area 3b is reactivated by inputs from the hand in roughly a normal somatotopic pattern. Quantitative measures of single neuron response properties reveal that reactivated neurons respond to tactile stimulation on the hand with high firing rates and only slightly longer latencies. We conclude that preserved dorsal column afferents after nearly complete lesions contribute to the reactivation of cortex and the recovery of the behavior, but second-order sensory pathways in the spinal cord may also play an important role. Our microelectrode recordings indicate that these preserved first-order, and second-order pathways are initially weak and largely ineffective in activating cortex, but they are potentiated during the recovery process. Therapies that would promote this potentiation could usefully enhance recovery after spinal cord injury.

## Introduction

The last 30 years of intensive research has led to a greatly improved understanding of how the somatosensory system of mature primates and other mammals responds to sensory loss, as reviewed by others (Buonomano and Merzenich, 1998; Jones, 2000; Wall et al., 2002; Kaas et al., 2008; Darian-Smith, 2009; Xerri, 2012). The types of sensory loss that have been experimentally studied have varied, ranging from the loss of peripheral nerves, especially the median nerve to the glabrous skin of the hand (Merzenich et al., 1983a,b; Silva et al., 1996; Florence et al., 1998; Xu and Wall, 1999), the sectioning of dorsal roots of peripheral nerve afferents as they enter the spinal cord (Pons et al., 1991; Jones and Pons, 1998; Darian-Smith and Brown, 2000; Darian-Smith, 2004; Darian-Smith and Ciferri, 2005, 2006), the loss of a digit or part of forelimb (Kelahan and Doetsch, 1984; Crockett et al., 1993; Florence and Kaas, 1995; Sengelaub et al., 1997; Florence et al., 1998; Li et al., 2013), the section of peripheral nerve afferents as they travel in the dorsal columns of spinal cord (Jain et al., 1997, 1998, 2000, 2008; Weng et al., 2003; Graziano and Jones, 2009; Qi et al., 2011a), or spinal cord injury in rodents (Ghosh et al., 2009; Aguilar et al., 2010; Humanes-Valera et al., 2013). All these types of sensory loss deactivate topographically matched parts of the somatosensory system, including parts of the ipsilateral dorsal column-trigeminal complex in lower brainstem and the contralateral ventroposterior nucleus (VP) of the thalamus, and the primary (area 3b) and other areas (1 and 2) of contralateral somatosensory cortex. These extensive deactivations were expected from the somatotopic organization and hierarchical arrangement of these processing stations.

What was not expected, at least by most investigators, was that over various amounts of time, the deactivated parts of the somatosensory system became responsive to tactile stimulation again, based on the somatosensory inputs that remained. Some changes in the receptive fields of neurons in the somatosensory system were apparent as soon as they could be measured after sensory loss (Wall, 1977; Calford and Tweedale, 1988; Faggin et al., 1997; Xu and Wall, 1997, 1999; Krupa et al., 1999), and these immediate changes could be attributed to the removal of the excitatory drive on central inhibitory neurons that normally constrain the receptive fields of relay neurons by keeping some of the excitatory inputs subthreshold. Other reactivations with greater changes in the receptive field locations of central neurons occurred over hours to weeks, such as the reactivation of cortical neurons previously activated from the glabrous skin of the hand by touch on the back of the hand after sectioning of the median nerve to the glabrous skin of digits 1–3 (Merzenich et al., 1983a,b). Such rapid reactivations may largely result from the potentiation of previously existing subthreshold inputs, especially in the cuneate nucleus representing the hand in the lower brainstem, by homeostatic mechanisms (Garraghty et al., 1991; Turrigiano, 1999; Wellman et al., 2002), and possibly by new axon growth over short distances (Darian-Smith and Gilbert, 1994; Jain et al., 2000; Darian-Smith, 2004; Hickmott and Steen, 2005; Cheetham et al., 2008; Yamahachi et al., 2009; Marik et al., 2010, 2014). Other reactivations that follow the loss or denervation of the forelimb, including invasion by inputs from the face (Pons et al., 1991; Jain et al., 1997, 2000; Wu and Kaas, 1999; Florence et al., 2000), may take many months to emerge (Jain et al., 1997), and depend on longer distances of new axon growth at subcortical and cortical levels (Florence et al., 1998; Jain et al., 2000). Cortical reorganization after limb amputations in humans can be found in several studies (Flor et al., 1995, 1998; Elbert et al., 1997; Davis et al., 1998; Lotze et al., 1999; Brugger et al., 2000; Moore et al., 2000; Curt et al., 2002). Thus, reactivations commonly occur in humans and other primates after a total loss of a subset of peripheral nerve inputs. In part, the reactivations after injury involve activity-dependent homeostatic cellular mechanisms, but also require the growth of new connections for greater changes in somatotopy. Because of the need for longer axon growth, greater changes in somatotopy take a longer time to emerge. Finally, somatotopic reorganization in cortex may usefully compensate for some of the sensory loss, but extensive reorganizations appear to lead to persisting mislocalization of sensory stimuli, such that the activation of hand cortex by inputs from the face leads to sensation felt on a phantom hand (Ramachandran, 1993; Davis et al., 1998; Ramachandran and Rogers-Ramachandran, 2000). Hence, somatosensory reactivations that restore much of normal somatotopy may be the most useful in restoring lost functions after sensory system damage. For example, highly somatotopic reactivations may occur when a loss of peripheral nerve afferents from any given region of skin is incomplete, as the subsequent potentiation of remaining weak or subthreshold inputs allows aspects of normal somatotopic order in central representations to be restored, at least partially. This may happen after selective sections of the dorsal roots of peripheral nerves as they enter the spinal cord (Darian-Smith and Brown, 2000; Darian-Smith and Ciferri, 2005, 2006), as similar inputs from a region of skin may enter over several dorsal roots (Welker, 1973), including roots left intact. In a similar manner, sectioning of peripheral nerve afferents as they ascend in the dorsal column of the spinal cord may leave a scattering of afferents that can restore some of the normal somatotopy of the deactivated somatosensory cortex (Jain et al., 1997). Alternatively, the information provided by cutting dorsal column afferents may not be totally lost, because these afferents branch as they enter the spinal cord. One branch ascends in the dorsal columns and the other terminates on the dorsal horn neurons in the spinal cord. As this sensory information is preserved, a more useful somatotopic reactivation of cortex may emerge based on the connections of the spinal cord neurons. The remainder of this review mainly focuses on the consequences of dorsal column lesions of the spinal cord in primates, including characteristics of natural recoveries and potential for augmented recoveries, but it is not intended to be a comprehensive literature review of other types of injuries and recovery mechanisms.

## Dorsal column lesions in primates

Dorsal column lesions in monkeys and other primates provide a number of advantages in studies of central nervous system reorganization after sensory loss. Most importantly, the loss is selective for the afferents that convey low-threshold, rapidly conducting tactile inputs from the slowly adapting and rapidly adapting receptors in the skin, while less rapidly conducting afferents for pain, temperature, and crude touch are left intact, as these afferents bypass the dorsal columns (Kaas, 2012). In addition, proprioceptive information from receptors located in muscles and joints travels in peripheral nerve afferents that ascend lateral to the dorsal column afferents in the spinal cord, where they are damaged only if the dorsal column lesion includes the fibers of the lateral spinal cord. Furthermore, the peripheral nerve afferents that contribute to the dorsal columns branch, such that one branch terminates in the dorsal horn of the spinal cord while another branch ascends in the dorsal columns to terminate in the brainstem. Although one branch is cut by the dorsal column lesion, the other branch may still function in the dorsal horn, where the afferents continue to contribute to spinal cord reflexes and other spinal cord functions, and possibly to ascending sensory pathways that compensate for dorsal column lesions. Studies of the effects of dorsal column lesions in rats have produced useful information (for reviews, see Kaas et al., 2008; Onifer et al., 2011); however, unlike the separation between the sensory and motor pathways in primates, corticospinal (motor) projections course in the dorsal columns in rodents (Hicks and D'Amato, 1975; D'Amato and Hicks, 1978; Vahlsing and Feringa, 1980; Paxino and Watson, 2007). Therefore, dorsal column lesions in rodents involve a direct motor loss in addition to the sensory loss. Thus, the use of dorsal column lesions in primates has the advantages of producing a rather specific loss of tactile capacities, while leaving other functions intact. Importantly, these specific lesions in primates are followed by unexpected levels of behavioral recovery (see below). While the lesions do not mimic the typical types of injury that occur in most cases of spinal cord injury in humans, where damage may be extensive and somewhat non-specific, the lesions can be made with precision and consistency so that useful results can be obtained from a few subjects. Consistent results from similar lesions allow therapeutic procedures to be evaluated. Thus, experimental results from monkeys have the potential of providing information that can usefully guide the interpretations and predictions of consequences of spinal cord damage in humans, and hopefully guide the development of therapeutic procedures.

**Figure 1:**
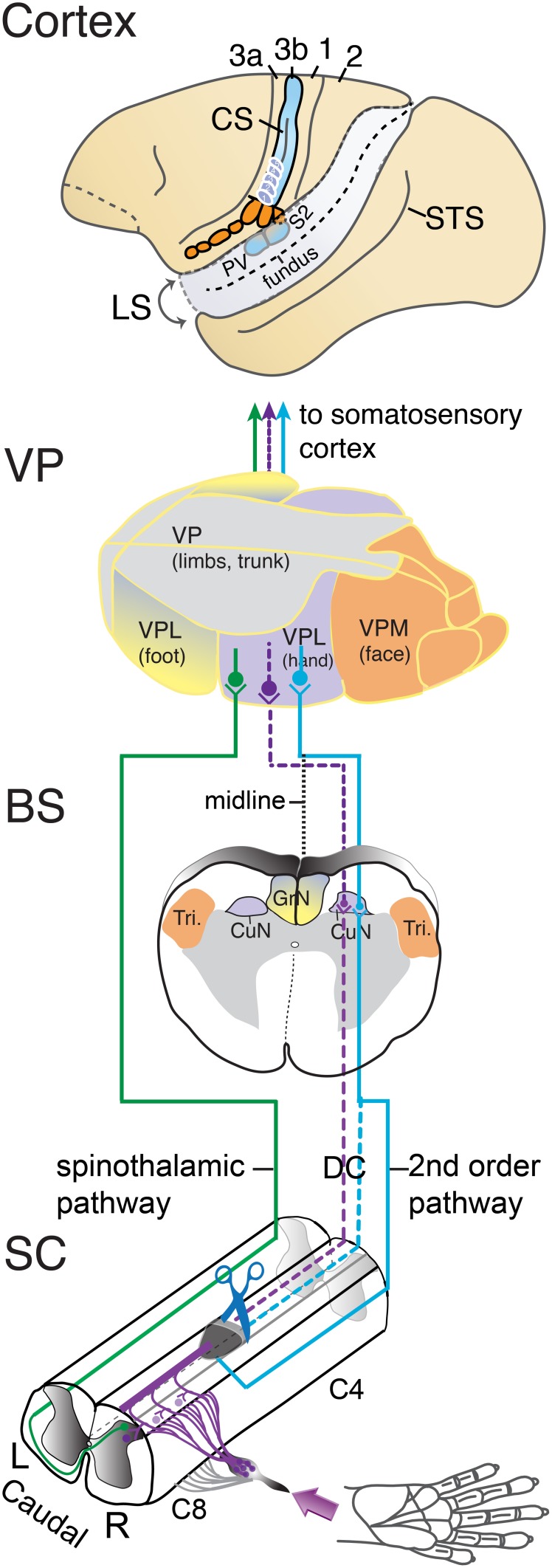
**Somatosensory pathways from the spinal cord to cortex of a squirrel monkey**. Cutaneous afferents from the hand that enter the cervical spinal cord between C4 and C8 levels via the dorsal roots are shown in the lower panel. In the spinal cord (SC), one branch of the afferents enters the ascending dorsal column pathway to terminate in the ipsilateral cuneate nucleus (violet line). The other branch terminates in the dorsal horn of the spinal cord. Second-order neurons activated in the dorsal horn by cutaneous primary afferents project to the ipsilateral cuneate nucleus via dorsal column, the dorsolateral pathway, or other pathways (blue line). Other dorsal horn neurons send axons to the contralateral spinal cord and form the spinothalamic pathway (green line). Cutting (scissors) the dorsal column pathway at a C4–C5 level deactivates the cuneate nucleus by removing the direct primary afferent inputs from the hand. The dashed lines mark the partially deafferented pathways from spinal cord to cortex after the spinal cord lesion. Most of the afferents of second-order tactile neurons in the dorsal horn that course in the dorsal columns may be preserved as well, but others may join the dorsal columns above the lesions, or travel outside the lesion in the dorsal lateral fiber tracts. The spinothalamic afferents may contribute to the reactivation of the ventroposterior nucleus and somatosensory cortex. The location of the elongated cuneate nucleus at the junction of the upper spinal cord with the lower brainstem (BS) is shown to represent how the pathways for touch on the hand travel. CuN, cuneate nucleus; Tri, trigeminal nucleus; ECN, external cuneate nucleus of the contralateral (left) somatosensory thalamus. In the somatosensory thalamus (VP), the larger ventroposterior lateral division, VPL, contains subnuclei for the hand and foot, which is capped by a region for the upper (proximal) portions of the limbs and trunk. The medial division, VPM, separates the face. Inputs from the hand are relayed to the hand subnucleus of VPL. The spinothalamic pathway terminates in the ventroposterior inferior nucleus (not shown) as well as VP. In cortex, somatosensory areas of the left cerebral hemisphere are shown. Squirrel monkeys have a short, shallow central sulcus, CS, so that the primary cortical area for touch, area 3b of Brodmann, is largely exposed on the brain surface. The region of the hand representation is indicated below the central tip of the central sulcus. Area 3b receives tactile information from VP. Area 1 contains a higher-order representation of touch just caudal to area 3b. Area 3a receives proprioceptive information from the ventroposterior superior nucleus of the thalamus (not shown). Area 2 is a higher-order somatosensory area just caudal to area 1. The second somatosensory area, S2, and the posterior ventral area, PV, are located on the upper bank of the lateral sulcus, LS. The superior temporal sulcus, STS, is shown for reference.

Our experiments typically involve a unilateral lesion of the dorsal columns at a C4–C5 level of the spinal cord (Figure 1). This removes the primary afferent tactile inputs to the cuneate nucleus from one hand, while leaving the inputs from the other hand intact. All inputs to the dorsal horn neurons of the spinal cord are preserved, as are the primary proprioceptive afferents in the lateral spinal cord pathway. Neurons in the elongated cuneate nucleus project to the hand and forelimb subnucleus of the ventroposterior nucleus (VP) of the contralateral thalamus. VP is commonly divided into a lateral “nucleus” (VPL) representing the contralateral upper and lower body, and a medial “nucleus” (VPM) representing the contralateral face and both the contralateral and ipsilateral tongue and teeth (Kaas et al., 2006). VP projects to primary somatosensory cortex, area 3b, in a somatotopic pattern, so that the hand subnucleus projects to the hand representation in area 3b just medial to that of the face and lateral to that of the arm and trunk. The VP inputs activate area 3b neurons, while VP provides subthreshold projections to area 1 (e.g., Nelson and Kaas, 1981; Garraghty et al., 1990a), which has a somatotopic organization that parallels and mirrors that in area 3b (Kaas et al., 1979).

In our experiments, we typically determine the effects of dorsal column lesions by recording from the hand subdivision of contralateral area 3b in squirrel monkeys or owl monkeys with microelectrodes. Squirrel monkeys have only a short, shallow central sulcus, and owl monkeys have only a short central dimple. For both of these primates, the representations of the hand in area 3b and in adjoining areas 3a and 1 are exposed on the surface of the brain. This exposure of the cortical areas allows activity patterns to be mapped in detail with densely spaced microelectrode penetrations. Microelectrode mapping can also be done at thalamic and brainstem levels, but not under visual guidance. Therefore, the somatotopy of these subcortical representations is both harder to determine in detail, and harder to illustrate compared to the two-dimensional cortical maps. In addition, reorganized area 3b maps reflect both subcortical alterations and those that originate in area 3b. Thus, area 3b is a key location for investigation in most experiments. We and others have also studied area 3b reorganization in macaque monkey (Pons et al., 1991; Florence and Kaas, 1995; Florence et al., 1998; Darian-Smith and Brown, 2000; Jain et al., 2008), where area 3b is hidden on the caudal bank of a deep central fissure, as in humans. These more difficult studies are important because macaques are used as a more common model for human brain organization, as macaque brains have some features of the somatosensory system that more closely resemble those of humans.

Neurons in the hand portion of area 3b typically have small excitatory receptive fields, mostly located on the glabrous skin of the hand, especially on the distal phalanges of digits, and they are often confined to part of a single phalange of a digit or pad of the palm. Nearby neurons have similar receptive fields, and a systematic partial map of the hand representation in area 3b can be reconstructed from a dense array of microelectrode recordings across the hand region by outlining all electrode penetration sites with receptive fields centered on any digit phalanges or pad of the palm, or part of the body (Merzenich et al., 1978; Nelson et al., 1980; Sur et al., 1982; Krubitzer and Kaas, 1990). Such maps are extremely consistent in somatotopy across individuals, and they conform to the orderly arrangement of projections from VP to area 3b (Jones et al., 1982; Kaas et al., 1984; Cusick and Gould, 1990; Qi et al., 2011b; Liao et al., 2013). In addition, narrow cell-poor septa separate the representations of digits in area 3b so that digit territories are histologically visible (Qi and Kaas, 2004). The most apparent of these cortical septa separates the representation of digit 1, the thumb, from that of the face; and this septum serves as a highly useful landmark when recording sites are related to cortical histology (Jain et al., 1998, 2001).

## The reactivation and reorganization of somatosensory cortex after dorsal column lesions

As the primary somatosensory cortical area for tactile stimuli, area 3b has been the most studied area after dorsal column injury. Most of the results have been obtained from multiple microelectrode recordings where receptive fields for cortical neurons have been defined by stimulating the skin with hand-held probes. Traditionally, near-threshold skin indentations are used to define the “minimal excitatory receptive field” (Merzenich et al., 1983a). In reactivated regions of cortex deprived of their normal sources of activation, neuron responses are subjectively classified as good or excellent, meaning that the neuron responses to light touch closely resemble those in normal cortex, or as poor, or non-responsive. Recordings in deprived cortex immediately after a dorsal column lesion reveal that this cortex is totally unresponsive to light tactile stimuli. Thus, no neuronal spikes are evoked in deprived cortex, while neurons in un-deprived cortex, such as face cortex, respond normally. After a dorsal column lesion at a high cervical level above C4, the hand cortex in area 3b is completely deprived and unresponsive. Over a period of 3–4 weeks, much of the deprived hand cortex starts to respond to touch on the hand, with a distorted but crudely normal somatotopy. Thus, neurons responsive to touch on digit 1 (thumb) are in cortex lateral to those for digit 2, and digits 1–5 are represented in a lateral to medial order. However, this pattern is often disrupted by displaced islands of neurons that represent the same digit, patches of cortex that remain unresponsive, and neurons with discontinuous receptive fields that represent more than one location on the hand. Such results have been reported in a number of studies, including those from owl monkeys, squirrel monkeys, marmosets, and macaque monkeys (Jain et al., 1997, 1998, 2008; Qi et al., 2011a; Bowes et al., 2012, 2013).

**Figure 2:**
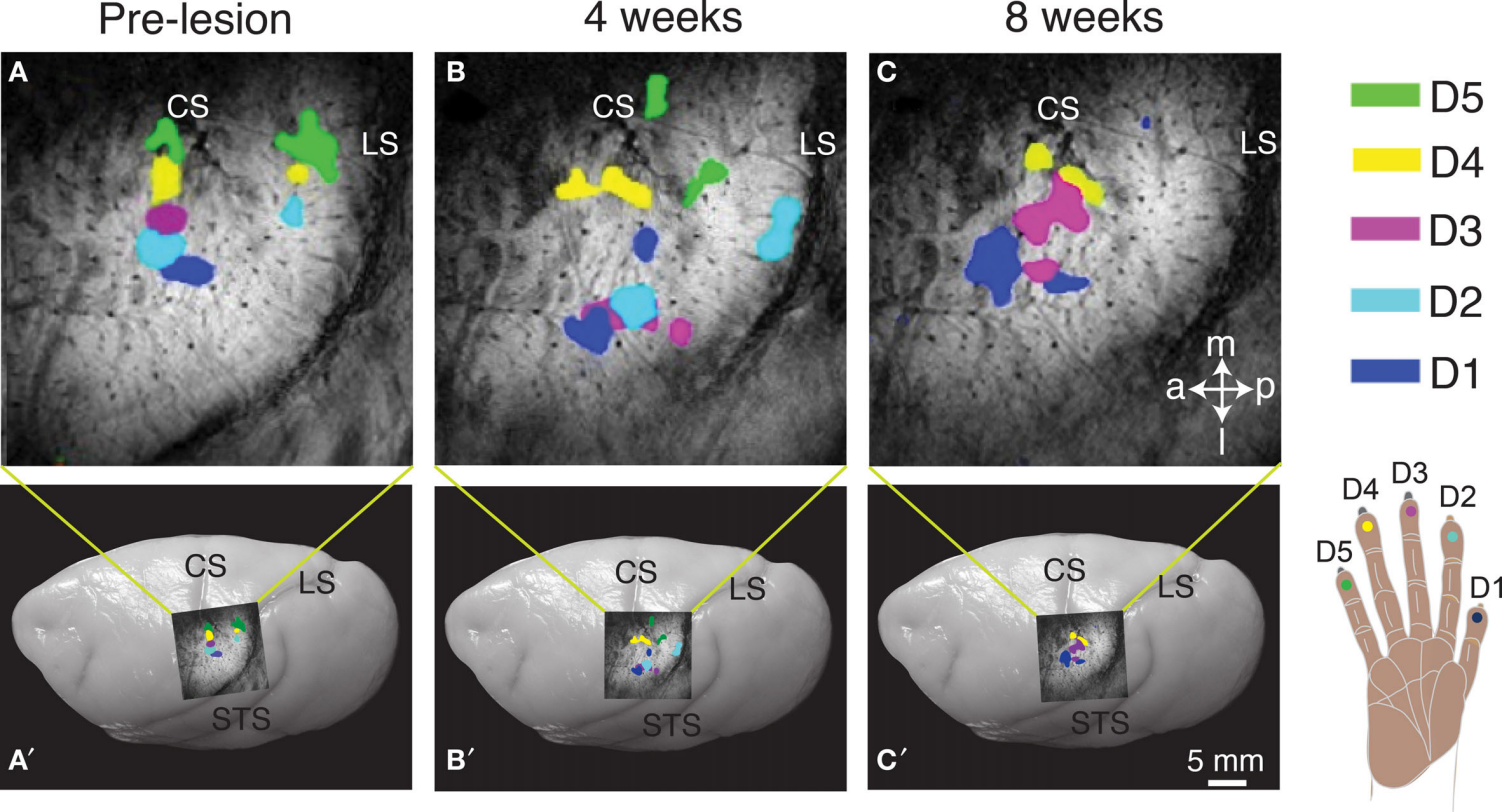
**Longitudinal mapping of fMRI activation to vibrotactile stimulation on digits 1–5 pre-lesion (A) and after 4-weeks (B) and 8-weeks (C) post-lesion following unilateral dorsal column section in a squirrel monkey (SM-O)**. Activation foci from trial blocks of stimulation on individual digits were superimposed for the composite images **(A–C)**. The location of the top panel on the brain is shown for each panel below. Dots on the hand schematic (lower right) are color-coded to represent the activation in cortex when that digit location was stimulated **(A–C)**. Superimposed images of the fMRI activation on photograph of a squirrel monkey brain indicates the approximate location of the region of interest **(A′–C′)**. Abbreviations: a, anterior; CS, central sulcus; D1–5, digits 1–5; l, lateral; LS, lateral sulcus; m, medial; p, posterior; STS, superior temporal sulcus. Adapted from Chen et al. (2012).

Studies of the reactivation process in monkeys with dorsal column lesion using imaging methods, such as fMRI, optical imaging, and radiographic imaging, have been limited but informative (Tommerdahl et al., 1996; Chen et al., 2012; Dutta et al., 2013; Yang et al., under revision). As an example, results from one squirrel monkey from Chen et al. (2012) are shown in Figure 2. Before a dorsal column lesion, tactile stimulation of each of the five digits resulted in a focus of activity in the hand region of area 3b, with foci for digits 1–5 in the expected lateral to medial order (Figure 2A. In this anesthetized monkey, responses in area 1 were not reliably obtained. Four weeks after a dorsal column lesion at the C4–C5 level, area 3b was responsive to touch on the digits, and the somatotopic order of foci activated from each digit was roughly in the normal order, but distorted from the normal pattern (Figure 2B). After 8 weeks, the cortex was more responsive, and the somatotopic pattern appeared more normal (Figure 2C). When the final somatotopic pattern from fMRI was compared to an optical imaging pattern and a microelectrode map in the same monkey, there was good agreement between the activation patterns from the three methods, but optical imaging produced a more detailed map than the fMRI imaging, and greatest detail was provided by the microelectrode maps. Although the fMRI mapping provides less sensitive somatotopic detail than the microelectrode recordings or the optical imaging, fMRI allows the same cortex to be imaged multiple times non-invasively. Over an eight-week or even longer period of recovery, there was no evidence that the deprived hand cortex was reactivated by any other part of the body, including the face. However, after longer periods of 6–8 months to years, face inputs from the region of the chin often activate parts of the hand cortex (Jain et al., 1997).

**Figure 3:**
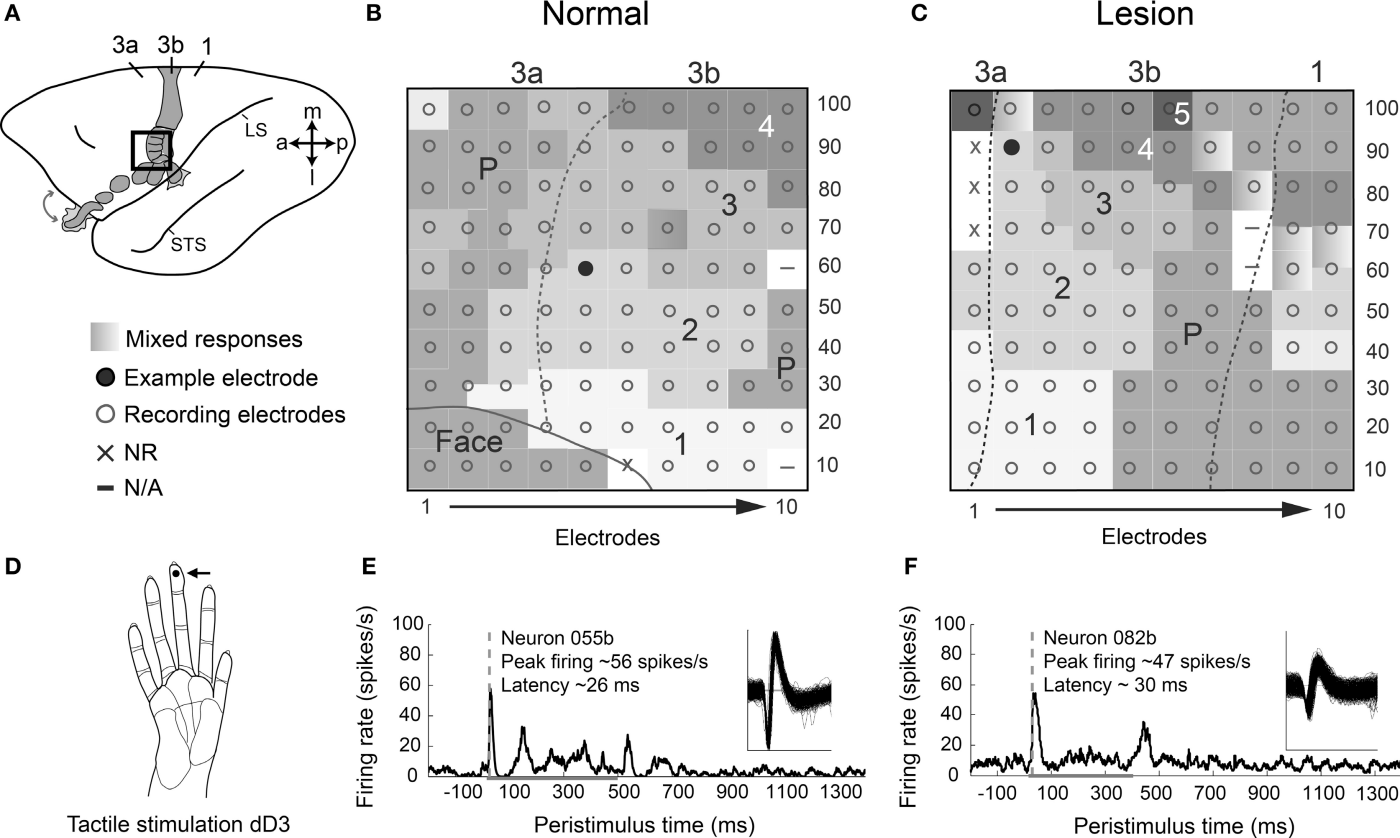
**Response properties of area 3b neurons after behavioral recovery from dorsal column lesion are similar to those in a monkey with no lesion. (A)** A schematic of the left hemisphere of an owl monkey brain with area 3b shaded shows the approximate placement of a 100-electrode array for monkey OM-A with no lesion (see **B**). The array placement for a monkey SM-G with a dorsal column lesion was similar, but slightly more medial and posterior (see **C**). Note the key for the somatotopic mapping of the array applies to **(B)** and **(C)**. **(B)** Representation of coverage of 100-electrode array in cortex for a normal monkey with somatotopic map reconstructed from locations of receptive fields for neurons at each electrode. Coverage included part of the area 3b hand and face representation as well as area 3a, with somatotopy consistent with previous reports from normal monkeys. The electrode location from which the example neuron for the lower panel **(E)** was recorded is indicated by the filled circle. **(C)** Representation of coverage of 100-electrode array in cortex for monkey SM-G after behavioral recovery from partial DC lesion at the C6 spinal cord level. This lesion preserved many of the dorsal column afferents from digits 1 and 2. SM-G experienced sensorimotor training and testing using the impaired hand for 6 weeks following DC lesion prior to recording with the multi-electrode array. All digits are represented within expected somatotopic locations, but D1 and D2 appear to be represented in more territory than D3–D5. Electrode location for the example neuron in the lower panel **(F)** is indicated by the filled circle. **(D)** Activities of neurons from monkeys with and without a lesion in **(B–C)** are shown in **(E–F)** during tactile stimulation on right distal digit 3 (dD3). The stimulation site is indicated by a dot (see arrow) on drawing of a monkey hand. **(E)** Example of a single unit 055b shows typical peak firing rates and latencies for an example normal case, monkey OM-A. Spikes during 100 trials of tactile stimulation on dD3 are averaged in the peristimulus-time histogram (PSTH). Waveforms of the recorded unit are shown (inset). **(F)** Example of a single unit 082b shows typical responses for a case with a dorsal column lesion, monkey SM-G, following recovery of reaching behavior with the impaired hand. Activity during 150 trials of tactile stimulation on dD3 is shown in the PSTH. In **(B–C)**, dashed lines indicate area 3b borders based on receptive field topography and myelin architecture. In **(E–F)**, dashed gray line indicates the latency of the peak firing response that exceeds 2 times the standard deviation of the average baseline firing. Peak firing value listed is baseline-subtracted. Horizontal gray line indicates stimulus duration in each trial (500 ms in OM-A, 400 ms in SM-G). Abbreviations: 1–5, digits 1–5; 3a, 3b, 1, area 3a, area 3b, area 1; a, anterior; dD3, distal digit 3; l, lateral; LS, lateral sulcus; m, medial; p, posterior; STS, superior temporal sulcus. Modified from Reed et al. (2010) and Qi et al. (2014).

When the dorsal column lesion is incomplete, perhaps as an unintended result, or because the lesion was intentionally placed at a lower C5–C6 level in order to preserve some inputs from digit 1 and a few from digit 2, then these preserved inputs come to represent larger than normal territories in area 3b after 5–8 weeks of recovery with sensorimotor training and testing. However, much of the activation is in the normal territories for those digits. Somatotopic organization of reactivated cortex after recovery is likely dependent on the level and extent of the lesion, as indicated by studies using single microelectrodes to map the cortex (Qi et al., 2011a; Chen et al., 2012) and using multi-electrode arrays implanted in cortex (Qi et al., 2014). For example, receptive field mapping of neurons recorded with a 100-electrode array in area 3b in a squirrel monkey showed that somatotopic organization in reactivated cortex was largely normal after behavioral recovery from a dorsal column lesion at the C6 level (compare Figures 3B,C). However, the cortical territories occupied by spared inputs for digits 1 and 2 encroached into regions deprived of input by the C6 lesion (digits 3–5). This monkey received sensorimotor training and testing for use of the impaired hand over the course of 6 weeks prior to recording with the array, and it is unknown what effect this behavioral intervention may have had on the cortical reactivation beyond what would have occurred spontaneously. In this case, preserved inputs activated their normal locations in cortex, but also came to activate larger than normal territories.

Finally, the reactivation of area 3b hand cortex should result in the reactivation of the hand territories of other cortical areas that depend on area 3b for activation, such as areas 1, S2, and PV, as reported previously (Pons et al., 1987; Burton et al., 1990; Garraghty et al., 1990b). Indeed, the area 1 representation of touch is restored in a somatotopic pattern that generally parallels that in area 3b, but it is less precise in detailed somatotopy, and the neuronal responses tend to be weaker in the reactivated regions (Merzenich et al., 1983a; Jain et al., 2008; Qi et al., 2011a). Responses to tactile stimuli also return to areas 3a and 2 (Garraghty et al., 1990a; Bowes et al., 2013) and the hand region of areas S2 and PV (Pons et al., 1987, 1992; Burton et al., 1990; Garraghty et al., 1990b; Tandon et al., 2009; Wang et al., 2013; Yang et al., under revision). Areas 3a and 2 receive proprioceptive input from the ventroposterior superior nucleus of the thalamus (Kaas, 2012), so neurons in these areas may remain responsive to hand manipulation after dorsal column lesions that spare the proprioceptive axons in the lateral spinal cord. As all these areas recover responsiveness to tactile stimuli and provide direct and indirect inputs to motor and premotor cortex (Krubitzer and Kaas, 1990; Stepniewska et al., 1993; Disbrow et al., 2003; Fang et al., 2005; Qi et al., 2010; Kambi et al., 2011; see Wise et al., 1997 for review), considerable sensory guidance of motor behavior is restored.

## The response properties of reactivated neurons in area 3b

Reactivated cortical neurons may have disorganized excitatory receptive fields, or larger than normal receptive fields, but these responses to light touch on the hand appear to range from subjectively normal amplitude to weak responses (Qi et al., 2014). Over recovery periods longer than 4–8 weeks, more neurons likely become capable of stronger responses. Quantifying response properties beyond receptive field organization is a growing focus of study in the somatosensory system (Doetsch et al., 1996; Wang et al., 2013; Qi et al., 2014), but some support for these studies comes from studies of reactivated neurons in primary visual cortex after retinal lesions (Chino et al., 1995, 2001; Darian-Smith and Gilbert, 1995). Our quantitative studies of the response properties of reactivated cortical neurons after dorsal column lesions have been limited (Wang et al., 2013; Qi et al., 2014), but there is already evidence that reactivated neurons have responses to tactile stimuli (indentation of the skin with an electromechanical probe) that are in the normal or near-normal range, as indicated by examples of cortical activity (Figure 3) from an intact monkey (Reed et al., 2010) and a monkey 6 weeks after mid-cervical dorsal column lesion (Qi et al., 2014).

Qi et al. (2014) found that fewer neurons in area 3b responded to tactile stimulation on the hand in monkeys after sensory loss followed by behavioral training compared to normal monkeys. However, peak firing rates of responsive neurons were similar in monkeys with and without lesions. An example neuron from a normal monkey (Figure 3E) maintained low levels of spontaneous firing and increased firing to about 56 spikes/s in response to the onset of a 500 ms depression of the skin on distal digit 3. This example showed some increased firing during the sustained indentation of the stimulus and a response to the removal of the stimulus from the skin. Such “off responses” are common in normal animals (Sur et al., 1984; Pei et al., 2009). The neuron's response with some maintained discharge during the duration of skin indentation is common in area 3b neurons, as these neurons can show mixtures of properties reflecting both rapidly-adapting and slowly-adapting afferents (Sur et al., 1984; Pei et al., 2009). The example neuron in the reactivated cortex after nearly complete dorsal column lesion (Figure 3F) responded to both stimulus onset and removal, while maintaining some firing during the sustained skin indentation on distal digit 3. Notably, the neuron in reactivated cortex had somewhat lower peak firing rates during tactile stimulation at about 47 spikes/s, but still greater than the median response rates reported previously (about 18 spikes/s) and seemingly close to rates expected normally (Reed et al., 2010; Qi et al., 2014). In 3 monkeys with dorsal column lesions and after hand use in a reaching task recovered to normal levels, response latencies of neurons in reactivated area 3b to skin indentation were similar to normal or slightly longer than normal, averaging about 31 ms [Reed et al., 2012 (abstract)]. Using similar recording methods and data analysis, Reed et al. (2010) found that response latencies of area 3b neurons in normal monkeys averaged about 21 ms. The example of neuronal activity in Figure 3 shows a neuron in a lesioned monkey with a response latency to skin indentation that was slightly longer than the latency of a normal neuron (compare Figure 3E to Figure 3F). Similar response rates with slightly longer latencies than normal may suggest that some sensory input reaches area 3b after dorsal column lesions through alternative pathways that require additional synapses to reach the target. More study is needed, but the reactivated neurons clearly have response properties that could usefully guide behavior.

## The effects of dorsal column lesion on behavior

There is no doubt that spinal cord injuries often have devastating consequences for functional outcomes. A large number of studies in rodents (e.g., Anderson et al., 2005; Courtine et al., 2008; Zorner et al., 2010) and monkeys (e.g., Schmidlin et al., 2011; Nout et al., 2012a,b) have investigated the behavioral deficits that occur after spinal cord injury. Other studies have focused on the effects of a restricted interruption of the ascending dorsal column somatosensory pathway on the dexterity of the forepaws in rodents (e.g., McKenna and Whishaw, 1999; Ballermann et al., 2001) and monkeys (e.g., Beck, 1976; Glendinning et al., 1992; Leonard et al., 1992; Cooper et al., 1993; Vierck, 1998; Vierck and Cooper, 1998; Qi et al., 2013). In humans and monkeys, the effects of dorsal column lesions on sensory behavior have been interpreted in two quite different ways in early reports. Thus, Rose and Mountcastle (1959) concluded that the “destruction of the posterior columns in man leads to a loss of the capacity to appreciate the position and movement of the limbs,” and “severe”—“disturbance in tactile sensations.” Later, Mountcastle further stated, “What remains in the mechanoreceptive sphere after… dorsal column lesion is the capacity to recognize that a mechanical stimulus has occurred; it is no longer possible to specify it exactly as to location, intensity or shape” (Mountcastle and Darian-Smith, 1968). These pronouncements clearly reflect what would be expected after damage of such a major sensory pathway. However, Wall (1970) quite differently concluded that “no sensory defect has been shown to follow isolated dorsal column lesion” in human, and “animals with complete dorsal column section can carry out discriminations of weight, texture, vibration, two points and position.” Later, Azulay and Schwartz (1975) summarized the effects of dorsal column lesion in monkeys by stating that “surprisingly little, if any, tactile functions are impaired after extensive damage to the dorsal funiculus.” Subsequent to these early reports, several studies have reported impairments of hand use after lesion of the dorsal columns in macaque monkeys (Glendinning et al., 1992; Leonard et al., 1992), and sensory alterations have been described in macaques (Vierck, 1973, 1974) and humans (Nathan et al., 1986).

**Figure 4:**
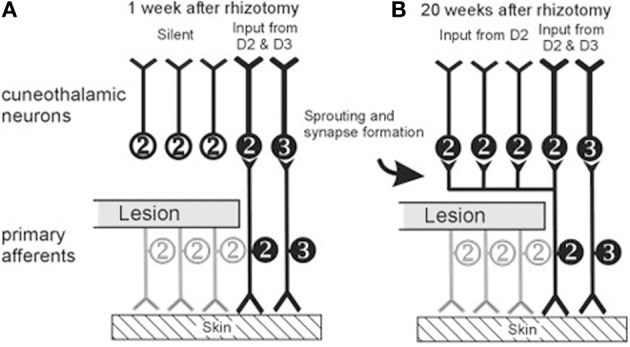
**Schematics represent hypothesized effects of a dorsal rootlet section that blocks sensory input from digit 1 (thumb) and digit 2 (index finger) in a macaque monkey after 1 week (A) and after 20 weeks (B)**. Inputs from digits 1 and 2 are labeled together as 2. After cutting the dorsal rootlet, some fraction of neurons innervating digits 1 and 2 may be spared, but a population of cuneate (and dorsal horn) neurons will be left without peripheral input. By 20 weeks after the dorsal rootlet section, terminals of the spared neurons innervating digits 1 and 2 have sprouted collaterals to innervate the previously deafferented target neurons in the cuneate nucleus. Similar mechanisms are hypothesized to occur at all levels of processing hierarchy from the brainstem to cortex. (From Darian-Smith, 2004, Figure 10, Copyright©2004 Wiley-Liss, Inc.. Reprinted with permission from John Wiley & Sons).

How can we explain these great differences in conclusions about the effects of dorsal column lesion in primates? There are several possible reasons for differences in findings and conclusions. (1) One possibility is that different lesions produce different results. For performance with the hand, the dorsal column lesion should be at the C4–C5 level or higher to remove all afferents from the hand, the lower lesions at C5–C6 of the cervical spinal cord spare some afferents from digit 1 and 2, and still lower lesions would spare inputs from most of the hand. Even with C4–C5 lesions or higher, various afferents in the dorsal columns could be spared. (2) Compensations likely occur. As described below for our study of the effects of dorsal column lesions on behavior, vision appears to supplement tactile feedback in food retrieval tasks after dorsal column lesions. Other compensations for sensory impairments are likely to be rapidly acquired as well. (3) Plasticity of the somatosensory system, even in mature primates appears to be a major source of recovery after dorsal column lesions. Recoveries of activation within the system and use of the hand may result from the potentiation of the activating effects of preserved dorsal columns afferents, even when they are very few. This possibility is well demonstrated by the effects of cutting most of the dorsal sensory roots of nerves of the forearm in monkeys, while leaving a few inputs from that hand that initially fail to activate cortex. However, several months later cortex responded to cutaneous stimulation of the largely deafferented digits. Figure 4 illustrates such an example reprinted from Figure 10 of Darian-Smith (2004; see also, Darian-Smith and Brown, 2000; Darian-Smith and Ciferri, 2005, 2006; Darian-Smith, 2009). This reactivation of cortex is due in part to the potentiation of preserved afferents likely through axon growth and the formation of new synaptic contacts on deactivated neurons occurring at brainstem, thalamic, and cortical levels (Darian-Smith and Gilbert, 1994, 1995; Florence et al., 1998; Jain et al., 2000; Darian-Smith et al., 2010). A related possibility is that after dorsal column lesion second-order spinal cord neuron inputs to the cuneate nucleus are also potentiated and thereby come to reactivate that nucleus by touch on the hand, effectively replacing some of the lost direct dorsal column inputs to the cuneate nucleus. We return to this possibility in the last section of this review. (4) Finally, some of the effects attributed to damage to the dorsal columns in previous studies may reflect damage to other afferents, as motor impairments may occur when the lesions extend into the lateral spinal cord where the dorsal lateral funiculus with proprioceptive afferents and the lateral corticospinal motor tract are located.

**Figure 5:**
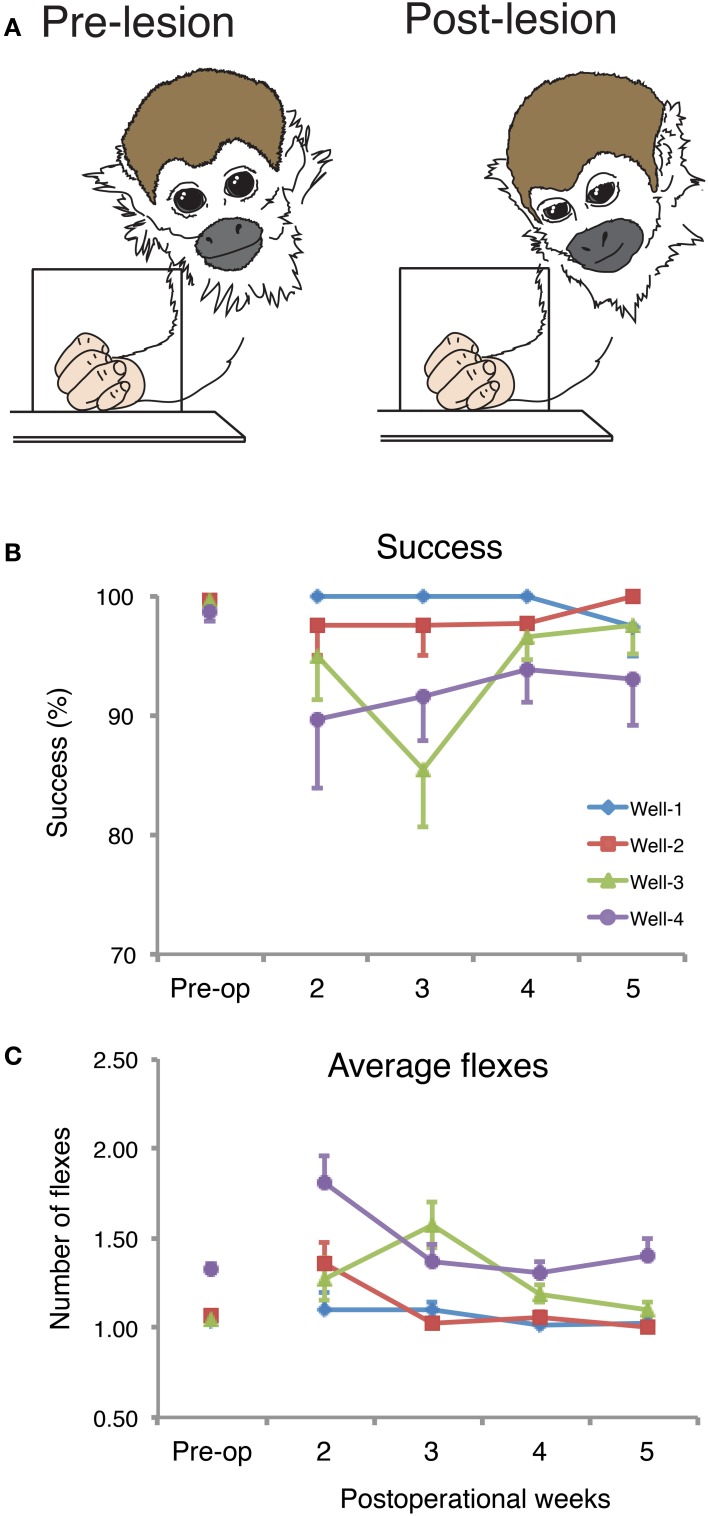
**Spontaneous recoveries after dorsal column injury can be incomplete or incorporate various compensations. (A)** Drawings from the captured photo-frames taken from a squirrel monkey (SM-O) during the reach-to-grasp task pre-lesion (left) and on post-op Day 15 (right). The schematic summarizes observations that after the spinal cord lesion, monkeys compensate for reduced tactile sensation by relying more often on vision. **(B)** Post-operational changes in the percentage of successful retrievals (mean, ± *SD*) in the reach-to-grasp task. Success scores in percent of the retrieval success for summed trials from each of the 4 testing wells per post-op week for monkey SM-O. Pre-op final week trials were summed. **(C)** Post-operational changes in mean number of flexes (mean, ± *SD*) for monkey SM-O. Each data point within each panel is the averaged number of flexes from the sum of the trials from each of the 4 wells over each testing week. Adapted from Qi et al. (2013).

The results of our recent study of hand use in squirrel monkeys after a unilateral dorsal column lesion provide evidence that the sensory loss produced by lesions confined to the dorsal columns do produce impairments in a food retrieval task (Qi et al., 2013). Monkeys were trained to reach for and retrieve small sugar pellets from wells that ranged from easy (shallow) to difficult (deep). Several results are presented in Figure 5. Before the spinal cord lesion, monkeys were very good at the task, even for the most difficult well. They quickly reached to grasp the pellet after briefly visualizing it, and retrieved it with one or sometimes 2 flexes of the digits to bring the pellet from the well to the mouth. When behavioral testing was continued two weeks after a complete or nearly complete lesion of the dorsal columns, the behavior was clearly impaired in that it took more than one attempt to successfully grasp objects from the difficult wells, and more flexes of the digits were needed. The video recordings of the task performance revealed another change in the behavior. Before the dorsal column lesion, the monkey looked at the pellet in the well only briefly as the monkey started the reach, and instead watched the investigator during the grasp and retrieval. This may seem strange, but extensive visual guidance was unnecessary, and the monkey may regard the investigator as a potential competitor for the food, as it would another monkey, or as potentially dangerous and in need of watching. In contrast, after the lesion, the monkey acted as if it could not sense by touch if the pellet was in the hand, and always looked to see if the pellet was there. During the retrieval, the palm was turned up so the presence of the pellet could be visually confirmed. Overall, we think that the deficits in the retrieval behavior were due to the sensory loss, as they occurred when the lesions did not include motor axons lateral to the dorsal columns. Early successes likely related to behavioral compensations, especially the visual assurance that the pellet had been grasped. In addition, sensory perception likely improved as early as 3–4 weeks after the lesion, as cortex became progressively more responsive to touch on the hand due to cortical reactivation.

## Mechanisms of cortical reactivation and the potential for therapeutic treatments

The reactivation of deprived hand cortex weeks to months after high cervical dorsal column lesion depends on the potentiation of preserved sensory pathways through axon growth and the formation of new synapses. We have previously suggested that dorsal column lesions are often incomplete, and that preserved but subthreshold dorsal column inputs to the cuneate nucleus gain strength by forming more synapses on more neurons as the result of reduced competition for synaptic space (Rasmusson and Northgrave, 1997; Xu and Wall, 1997; Darian-Smith, 2004; Darian-Smith and Ciferri, 2006). Similar changes likely occur at the levels of the ventroposterior nucleus and somatosensory cortex (Garraghty et al., 1991, 2006; Rasmusson, 1996; Wellman et al., 2002). Under conditions of extreme loss of afferents to the cuneate nucleus, we had evidence that even afferents from the adjacent trigeminal complex for the face could sprout and grow to innervate the cuneate nucleus (Jain et al., 2000), providing relays to the hand subnucleus of the contralateral ventroposterior nucleus and then to the hand territory of primary somatosensory cortex. While such reactivations of the cuneate nucleus by inputs from the hand that were preserved after the dorsal column lesion, as well as those that grow into the cuneate nucleus from the face, do occur, we have observed reactivations of hand cortex after lesions of the dorsal columns that were 95–100% complete (Qi et al., 2011a). Although it can be difficult to accurately determine the completeness of a lesion of the dorsal columns from histological sections through the lesion site, we feel confident about the completeness of experimental lesions because we injected tracers into the fingers of both hands in monkeys with unilateral lesions to demonstrate the loss of labeled axon terminations in the cuneate nucleus on the lesioned compared to the non-lesioned side (Qi et al., 2011a). Thus, we have strong evidence that there are reactivations that do not depend on preserved dorsal column first-order afferents, and thus depend mostly on other afferents from the hand.

The most likely alternative pathway is from neurons in the dorsal horn of the spinal cord that project to the dorsal column nuclei (Perl et al., 1962; Rustioni et al., 1979; Bennett et al., 1983). These neurons are directly activated by one of the bifurcated branches of peripheral nerve axons that terminates in the dorsal horn, while the other branch ascends in the dorsal columns and terminates in the dorsal column nuclei (Wall, 1970; Willis and Coggeshall, 2004). These dorsal column neurons would receive the same information for the skin of the hand that the cuneate nucleus directly receives, although there may be differences in the amounts of convergence of inputs and inhibitory mechanisms so that receptive field sizes may differ for neurons in the dorsal horn or cuneate nucleus (Dykes and Craig, 1998; Witham and Baker, 2011). However, the normal role of second-order spinal cord inputs to the cuneate nucleus most likely would be to provide subthreshold activation, because cortical reactivation after a loss of the primary inputs to the cuneate nucleus takes weeks to emerge.

In order to be effective, the projections to the cuneate nucleus by second-order dorsal horn neurons must survive the dorsal column lesion. Some axons from second-order neurons might enter the dorsal columns below the lesion and be lost. Others might enter above the lesion, or travel in some other pathway, such as the lateral funiculus, which is dominated by proprioceptive afferents (Rustioni et al., 1979). This possibility seems likely in that larger lesions of the dorsal column that extend into the lateral fiber tracts appear to reduce the extent of the reactivation of the hand cortex from inputs from the hand.

**Figure 6:**
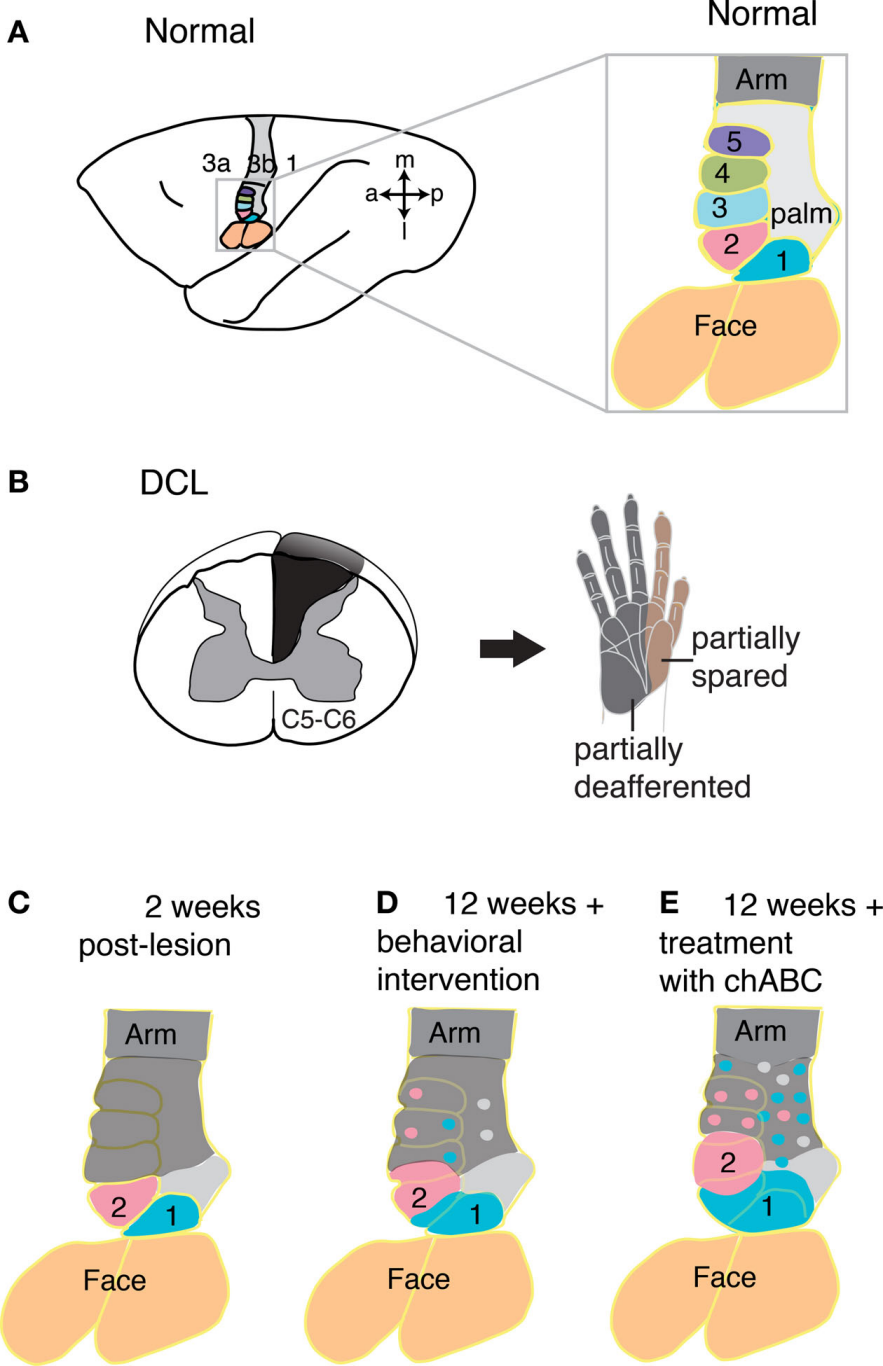
**Spontaneous and augmented recoveries from sensory loss in monkey cortex. (A)** Schematic view of the left hemisphere of an owl monkey brain with area 3b highlighted in color and expanded (right) to focus on the hand representation. **(B)** When afferents devoted to the hand are cut in the cervical spinal cord at the C5 or C6 level in squirrel monkeys and owl monkeys (left), inputs from much of digit 1 and digit 2 are spared, while inputs from most of the rest of the hand are disrupted, as indicated by the hand schematic (right). **(C)** After such a lesion **(B)**, the hand representation in primary somatosensory cortex is immediately rendered largely unresponsive to tactile stimuli (gray shading), with the exception of much of the digit 1 and digit 2 representations (blue and pink shading, respectively), for which the majority of axons enter the spinal cord above the level of the lesion. Over weeks of spontaneous post-lesion recovery without augmentation, the deactivated cortex becomes partly reactivated by inputs from the hand with activation from the spared dorsal column inputs (digits 1 and 2), and possibly other pathways (not shown). **(D)** This schematic summarizes expected results in monkeys with dorsal column lesions that received behavioral intervention when the cortex is studied 12 weeks after the lesion. Sensorimotor training and testing for use of the impaired hand appears to promote reactivation of cortex, but further study is needed to determine how effective behavioral intervention is beyond spontaneous recoveries and in comparison with other types of treatments. Based on previous findings and ongoing research, we expect that the representations of spared digits will expand their territories and islands of responsiveness will emerge in previously unresponsive zones. **(E)** The effectiveness of a few preserved dorsal column afferents for activating cortex can be enhanced by treatment of the target site in the cuneate nucleus with the enzyme chondroitinase ABC shortly after the spinal cord injury. Digestion of components of the extracellular matrix in the cuneate that inhibit axon sprouting allows axons spared from the lesion to sprout collaterals and innervate additional territory, which is reflected at the cortical level (examined 12 weeks after lesion and enzyme treatment). Thus, augmentation in this way produces expanded representations of the spared inputs from digits 1 and 2 in cortex, and possibly enhances ability of axons of second-order spinal cord neurons to sprout and innervate islands of the deprived cuneate nucleus. Abbreviations: 1–5, digits 1–5; 1, 3a and 3b, areas 1, 3a and 3b; a, anterior; C5–6, cervical spinal cord segments 5–6; chABC, chondroitinase ABC; DCL, dorsal column lesion; l, lateral; m, medial; p, posterior. Based on Jain et al. (1997), Bowes et al. (2012), and Qi et al. (2014).

Given the evidence that the reactivation of the cortex by inputs from the hand is important for the recovery of manual dexterity, what can be done to promote these reactivations? Recently, we have investigated two interventions for promoting reactivations. First, we found promising evidence that behavioral training after sensory loss improved the recovery of hand use in a retrieval task and correlated with cortical reorganization [Qi et al., 2010, 2012 (abstracts)]. Second, we found that an injection of an enzyme, chondroitinase ABC, into the cuneate nucleus after the lesion of the dorsal columns promotes the reactivation of hand cortex by preserved dorsal column afferents (Bowes et al., 2012). This enzyme digests chondroitin sulphate proteoglycans, which make up the extracellular matrix around neurons and glia. Treatment with chondroitinase ABC was first shown to promote recovery after spinal cord injury in rats by Bradbury et al. (2002). The extracellular matrix around neurons in the cuneate nucleus provide a chemical and physiological barrier that appears to inhibit new axon growth into and within the nucleus, thereby limiting the reactivation of deprived neurons in the nucleus, and the relay of driven activity to higher levels of the somatosensory system. By intentionally sparing dorsal column afferents from the thumb (digit 1) in squirrel monkeys by placing the dorsal column lesion at a lower cervical level (C6), and using the chondroitinase ABC enzyme injections near the targets for new growth from the spinal cord into the brainstem, more of the hand representation in contralateral somatosensory cortex was activated than in monkeys without this injection (Figure 6). Chondroitinase ABC has been used by others in a number of different experiments to increase axon sprouting, including the growth of dorsal column axons past a lesion site in rats (Massey et al., 2006, see reviews by Onifer et al., 2011; Garcia-Alias and Fawcett, 2012). Other promising treatments for recovery after sensory loss or spinal cord injury include the use of antibodies to a major myelin-associated neurite growth inhibitor, named Nogo-A (see Fawcett et al., 2012). The local placement of factors that promote growth and provide attraction to a target may also be useful (Schnell et al., 1994). Additional treatments for spinal cord injury have been reviewed by others (He and Koprivica, 2004; Buchli and Schwab, 2005; Bradbury and McMahon, 2006; Cafferty et al., 2008; Giger et al., 2010; Cregg et al., 2014; Silva et al., 2014). Sensorimotor training and testing for use of the impaired hand in monkeys (Qi et al., 2013, 2014) or impaired limbs in rodent (Kao et al., 2009; Krajacic et al., 2010; Graziano et al., 2013), appears to promote functional recovery and reactivation of cortex, but further study is needed to determine how effective behavioral intervention is beyond spontaneous recoveries or in relation to pharmacological treatments such as chondroitinase ABC. Combining pharmacological and behavioral interventions may promote even more useful recoveries in primates, as promising results have been reported in rodent models (Tetzlaff et al., 2009; Garcia-Alias and Fawcett, 2012; Zhao et al., 2013). In conclusion, we stress that much progress has been made over the last several years in understanding the recovery process that occurs after sensory loss following dorsal column injury. Much of this plasticity was unexpected, but the spontaneous recovery accounts for the early misinterpretations that the dorsal column pathway has little sensory significance. In addition to the unexpected spontaneous recovery, there are also hopeful signs that various therapeutic treatments have the potential of greatly improving the course of recovery.

## Author contributions

All authors contributed to the writing of the manuscript. The findings reviewed are those of the authors, as well as those of other investigators.

### Conflict of interest statement

The authors declare that the research was conducted in the absence of any commercial or financial relationships that could be construed as a potential conflict of interest.
